# Evaluation of deep eutectic solvents in the synthesis of molecularly imprinted fibers for the solid-phase microextraction of triazines in soil samples

**DOI:** 10.1007/s00216-024-05164-5

**Published:** 2024-02-03

**Authors:** Alexia Monnier, Myriam Díaz-Álvarez, Esther Turiel, Antonio Martín-Esteban

**Affiliations:** Departamento de Medio Ambiente y Agronomía, INIA-CSIC, Carretera de A Coruña Km 7.5, 28040 Madrid, Spain

**Keywords:** Green chemistry, Deep eutectic solvents, Molecular imprinting, Sample preparation, Solid-phase microextraction

## Abstract

Nowadays, molecularly imprinted polymers (MIPs) are well established and are considered excellent materials for performing selective extractions. However, with the progressive implementation of the principles of green chemistry, it is necessary to find greener alternatives for both the synthesis and further use of MIPs in sample preparation. Accordingly, in the present work, different deep eutectic solvents (DES, both hydrophilic and hydrophobic), as an alternative to conventional organic solvents (i.e., toluene), were evaluated as porogens for the synthesis of imprinted fibers (monoliths), using fused silica capillaries as molds, for solid-phase microextraction (SPME). From this study, the polymer prepared with propazine (dummy template), methacrylic acid (monomer), ethylene glycol dimethacrylate (cross-linker), and a formic acid:L-menthol (1:1) DES (porogen) showed the best performance for selective rebinding of triazines. After optimization of the different variables involved in SPME, the new imprinted fibers were successfully applied to the extraction of target analytes (desisopropylatrazine, desethylatrazine, simazine, and atrazine) from soil sample extracts, providing relative recoveries ranging from 75.7 to 120.1%, reaching limits of detection within the range of 6.2–15.7 ng g^−1^, depending upon the analyte.

**Graphical Abstract:**

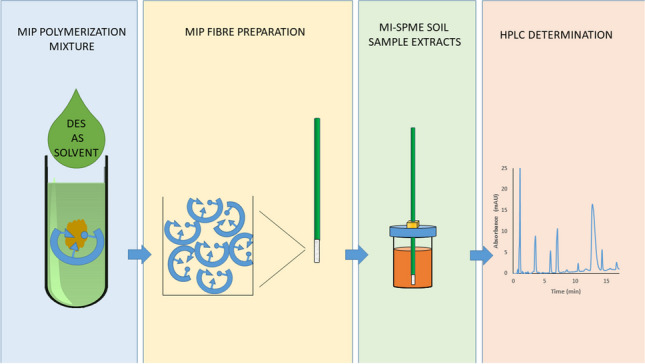

**Supplementary Information:**

The online version contains supplementary material available at 10.1007/s00216-024-05164-5.

## Introduction

In recent years, several new sorbent materials have been proposed for use in sample preparation to improve both the sensitivity and selectivity of the analytical process [1, 2]. Among them, molecularly imprinted polymers (MIPs), tailor-made materials able to provide selective extractions, have been extensively studied [3, 4] and successfully incorporated as sorbents in various (micro)extraction techniques [5, 6]. MIP synthesis is easy to perform in any laboratory without the need for sophisticated instrumentation. First, a template molecule is mixed with functional monomers in a suitable solvent (porogen) capable of maximizing template:monomer interactions (hydrogen bonding, van der Waals forces, etc.). A large amount of cross-linker is then added to the mixture and polymerization is carried out. After polymerization, the template molecule is removed, leaving cavities within the polymer network that are complementary in size, shape, and chemical functionalities. Accordingly, the resulting polymer is ideal for providing selectivity in sample preparation due to its inherent ability to recognize the template and related compounds in rebinding experiments using an appropriate solvent. However, such synthesis is not in line with the principles of green chemistry [7, 8], and almost every step of the MIP synthesis would have to be critically assessed in order to establish more sustainable alternatives. In fact, most of the monomers and cross-linkers (e.g., methacrylic acid, ethylene glycol dimethacrylate) typically used for MIP synthesis are considered harmful. In addition, large amounts of toxic solvents (e.g., toluene) are used, and replacing them with more environmentally friendly solvents would be desirable. Furthermore, MIP synthesis by bulk or precipitation polymerization (the most common polymerization strategies) generates a large amount of solid or liquid waste, respectively. In this context, the sustainability of MIP synthesis has been qualitatively assessed, and some guidelines for its improvement have recently been proposed [9–11].

Most of the MIPs reported in the literature are prepared following the so-called non-covalent approach, where the template and monomer interact with each other by hydrogen bonding, electrostatic interactions, and/or metal ion coordination, both in the pre-polymerization mixture and during rebinding experiments. For this purpose, solvents capable of stabilizing such interactions, such as chloroform and toluene, are recommended. In this regard, the solvent is responsible for maximizing the template–monomer interaction in the pre-polymerization mixture, and thus the selection of a proper porogen (solvent) is key in molecular imprinting. Furthermore, the porogen used is responsible for the formation of a proper porous structure to allow the diffusion of target analytes from the sample to the binding sites. However, from a green chemistry perspective, the use of the aforementioned toxic and harmful organic solvents should be minimized, and some greener alternatives have been proposed in recent years.

Ionic liquids (ILs), which are molten salts with melting points close to room temperature, have been considered as a class of green solvents due to their tunable properties, negligible vapor pressure, and non-flammability [12]. ILs have been used as porogens in the preparation of imprinted polymers, providing improved selectivity [13] and superior performance under polar conditions [14]. However, although ILs were initially considered as green solvents, it has been reported that ILs exhibit hazardous toxicity and poor biodegradability [15, 16]. As an alternative, deep eutectic solvents (DES) have been proposed as a new generation of green solvents [17]. DES are prepared by mixing two components (solid or liquid)—a hydrogen bond acceptor (HBA) and a hydrogen bond donor (HBD)—able to interact with each other through hydrogen bonding. The resulting DES are liquid at ambient temperature and nowadays are widely used in different fields [18–22]. However, the use of DES in molecular imprinting is still at an early stage of development, and DES have mainly been used as auxiliary solvents in combination with conventional solvents as porogens. In this regard, a DES synthesized using choline chloride (HBA) and glycerol (HBD) was combined with a methanol:water mixture for the synthesis of a MIP for the extraction of chlorogenic acid [23]. The obtained DES-based MIP exhibited higher affinity for the target analyte than both the corresponding DES-based NIP and the conventional NIP and MIP without the presence of DES.

Similarly, three different DES, using choline chloride in combination with ethylene glycol, glycerol, or propylene glycol, were studied as auxiliary solvents using acetonitrile as porogen in the synthesis of chloramphenicol-imprinted polymers for the selective pipette-tip solid-phase extraction of chloramphenicol from milk samples [24]. From this study, it was concluded that DES prepared with ethylene glycol yielded a MIP with the highest absorption capacity and improved mass transfer. The results indicated that the final performance of the obtained DES-based MIP is influenced by the components used in the preparation of the DES. The improved performance of the reported DES-based MIPs might be related to the presence of high-content functional groups in DES monomers, leading to enhancement of the selectivity and affinity of DES-based MIPs [25]. However, there is still a lack of studies to explain the reasons for the aforementioned improvements. Moreover, in most cases, DES have been used as one of the components of the porogen, and thus the role of DES in the performance of the resulting DES-based MIPs is rather unclear. In fact, the amount of DES present in the final porogen was less than 30% (v/v), and it is even questionable whether the pre-formed DES remains unaltered when mixed with other solvents (water, methanol, and acetonitrile) at such low levels. In this regard, it has been reported that above 51% of the hydration level, the DES–water mixture should be described as an aqueous solution of DES components, as the nanostructure of the DES is not retained at such dilution [26, 27]. Accordingly, further research in this area is needed in order to clarify the mechanisms underlying the observed improvements in DES-based MIPs.

Therefore, in the present work, different hydrophilic and hydrophobic DES were prepared and evaluated as porogen/monomer in the synthesis of molecularly imprinted fibers to be used in the solid-phase microextraction (SPME) of triazines, with the main aim of replacing conventional harmful solvents used in MIP synthesis with DES as a greener alternative. Variables affecting the synthesis of imprinted fibers (i.e., polymerization time) and the selective recognition of target analytes in organic solvent (toluene) were investigated and properly optimized. Using the optimal DES, composed of L-menthol and formic acid, the obtained fibers were able to selectively recognize several triazines in soil sample extracts.

## Materials and methods

### Chemicals and reagents

Desisopropylatrazine (DIA), desethylatrazine (DEA), simazine (SIM), atrazine (ATZ), and propazine (PPZ), isoproturon (IPN), methacrylic acid (MAA), ethylene glycol dimethacrylate (EGDMA), and 2,2′-azobis-2-isobutyronitrile (AIBN) were supplied by Sigma-Aldrich (Madrid, Spain). AIBN was recrystallized in methanol, and EGDMA and MAA were freed from the stabilizers by distillation under reduced pressure prior to use. Formic acid (FA), L-menthol, thymol, camphor, choline chloride (ChCl), and betaine hydrochloride (BetCl) were purchased from Merck (Madrid, Spain). High-performance liquid chromatography (HPLC)-grade acetonitrile (ACN), toluene, and acetone were obtained from Honeywell (Seelze, Germany). Purified water was obtained from a Milli-Q purification unit supplied by Millipore (Madrid, Spain). All other chemicals were of analytical reagent grade and were used as received. Stock standard solutions of triazines (1 g/L) were prepared in acetonitrile and kept at −22 °C. The chemical structures of the triazines and isoproturon are shown in Fig. S1.

### Preparation of deep eutectic solvents

L-Menthol, thymol, camphor, choline chloride, or betaine chloride, acting as HBA, was combined with a given HBD (MAA or FA) in a glass vial at different molar ratios, as shown in Table 1. The vial was tightly closed and placed in an oven at 60 °C equipped with a roller rotating at 24 rpm (Barloworld Scientific, Staffordshire, UK) for a given time (Table 1). After melting, the vial was allowed to cool to room temperature prior to further use.

**Table 1:** List of the DES prepared, indicating the molar ratio, experimental conditions, and their appearance after synthesis

HBD:HBA	Molar ratio	Experimental conditions (temperature, time)	Appearance at room temperature
MAA:L-menthol	1:1	60 °C, 15 min	Homogeneous liquid
MAA:camphor	1:1	60 °C, 15 min	Homogeneous liquid
MAA:thymol	1:1	60 °C, 15 min	Homogeneous liquid
MAA:ChCl	1:1	60 °C, 45 min	No formed
	2:1	60 °C, 15 min	Homogeneous liquid
MAA:BetCl	1:1	60 °C, 45 min	No formed
	2:1	60 °C, 60 min	Unstable homogeneous liquid
	3:1	60 °C, 60 min	Homogeneous liquid
FA:L-menthol	1:1	60 °C, 15 min	Two-phase liquid

Optimal DES was characterized by Fourier-transform infrared (FT-IR) spectroscopy on a Jasco FT/IR-460 Plus spectrophotometer. All spectra were recorded by attenuated total reflectance between 4000 and 400 cm^−1^, with the sample in the solid or liquid state without any other treatment.

### Preparation of molecularly imprinted fibers

Polymerization mixtures were composed of template molecule (PPZ, 0.15 mmol), functional monomer (MAA, 0.60 mmol), cross-linker (EGDMA, 3 mmol), initiator (AIBN, 0.13 mmol), and porogen (toluene or prepared DES, 0.87 mL). The direct preparation of MIP fibers (monoliths), using fused silica capillaries as molds, was performed according to the experimental procedure described elsewhere [28] and is depicted in Fig. S2. Briefly, it consists in filling a capillary (0.53 mm i.d.) with the polymerization mixture described above and performing polymerization in an oven at 65 °C for a given time (30, 60, 120, and 150 min). After polymerization, the capillaries were cut and silica was etched away by immersion in a 3 M aqueous solution of NH_4_HF_2_ for 12 h under agitation. Finally, the template was removed by immersing the MIP fibers in a methanol:acetic acid solution (1:1, v/v) for 2 h. Non-imprinted polymer fibers were also prepared following the same experimental procedure but without the addition of the template. Following this procedure, MIP and NIP monoliths of 1 cm length and 0.53 mm thickness were obtained. An Olympus SZX12 microscope equipped with a digital camera was used for the acquisition of optical micrographs of the resulting fibers.

### Molecularly imprinted SPME procedure

The molecularly imprinted SPME (MI-SPME) procedure involved the conventional conditioning, loading, washing, and elution steps, and was adapted from [28]. Before loading, the fibers were immersed in toluene for 15 min. Then, fibers were immersed in 1.7 mL of standard (single analyte or mixtures of triazines for selectivity studies) or sample extract solutions in toluene for 30 min under stirring at 450 rpm using an orbital stirrer (Vibramax 100, Heidolph, Kelheim, Germany). After loading, and in order to remove nonspecific interactions, the fibers were washed by immersion in a toluene:acetonitrile mixture (97.5:2.5 v/v) for 15 min under stirring. Finally, the fibers were air-dried for 10 min to remove traces of toluene, and the target analytes were eluted with 225 µL of methanol in a 0.45 mL vial insert by stirring for 15 min. Methanolic extracts were diluted to 450 µL with water for HPLC analysis. Between samples, fibers were reconditioned by immersion in acetonitrile and toluene in two consecutive steps of 15 min each.

### Sample preparation

Soil samples were collected from an experimental plot located in the region of Madrid (La Canaleja, Alcalá de Henares). Soil samples were sieved (2 mm) and stored at room temperature until analysis. Soil sample extracts were obtained by ultrasonic-assisted extraction in small columns, as described elsewhere [29]. Briefly, 10 g of soil was placed inside a glass column equipped with a polyethylene frit at the bottom. After the addition of 20 mL of ACN, extraction was performed by sonication in an ultrasonic bath for 15 min. The sample extracts were then collected and filtered through a 0.45 µm polytetrafluoroethylene (PTFE) syringe filter (Scharlab, Barcelona, Spain). Finally, the extracts were evaporated to dryness under a gentle air stream and then reconstituted with 5 mL of toluene.

For validation purposes, soil samples were spiked with a mixture of triazines at different concentrations, ranging from 0.01 to 0.25 µg g^−1^, and extracts were obtained as described above. Then, MI-SPME was performed and the analytes were determined by HPLC with diode array detection (HPLC–DAD) at 220 nm. Calibration graphs were constructed for all the analytes within the concentration range indicated above. The limits of detection (LODs) and quantitation (LOQs) were calculated as three and ten times, respectively, the standard deviation of the lower concentration tested.

### Chromatographic analysis

All chromatographic measurements were performed using an Agilent Technologies 1200 Series HPLC instrument equipped with a quaternary high-pressure pump, vacuum degasser, autosampler, and diode-array detector. A sample volume of 100 µL was injected into a KromaPhase (100 mm × 4.0 mm i.d., 3.5 µm d.p.) analytical column (Scharlab, Barcelona, Spain), and the analytes were separated at 1 mL min^−1^ by gradient elution from 85% water (A) and 15% acetonitrile (B) to 50% A and 50% B in 19 min, returning to initial conditions in 3 min. Triazinic herbicides were monitored at 220 nm and quantified by external calibration using peak area measurements.

## Results and discussion

### Preparation of deep eutectic solvents and preliminary evaluation as component in the polymerization mixture

DES were prepared by combining ChCl, BetCl, L-menthol, camphor, or thymol as HBA with FA or MAA as HBD. The resulting DES were evaluated as porogens for the subsequent preparation of MIP fibers, while DES containing MAA were evaluated as porogens/monomers. As shown in Table 1, the different HBD:HBA molar ratios tested ranged from 1:1 to 3:1. In general, hydrophobic DES (based on L-menthol, camphor, and thymol) were easily obtained at a 1:1 molar ratio at 60 °C in 15 min and remained stable at room temperature for further use. On the contrary, hydrophilic DES (those based on ChCl and BetCl) required molar ratios higher than 1:1 and longer preparation times. Stable DES were preliminarily tested as porogen for the preparation of imprinted monoliths. During the preparation of the polymerization mixture, it was observed that the polymerization components (monomer and cross-linker) were not soluble in MAA:ChCl DES, thus preventing the subsequent polymerization. Alternatively, MAA:BetCl DES was able to dissolve all polymerization components, but after polymerization, the obtained fibers were more transparent and brittle than those obtained using hydrophobic DES regardless of polymerization time (30 and 60 min), suggesting a low degree of cross-linking. Figure S3 shows a microscopic image comparing the fibers obtained using MAA:BetCl and MAA:L-menthol DES as porogen. Nevertheless, MAA:BetCl fibers were tested in rebinding experiments with atrazine standard solutions in toluene. After a washing step as described in the experimental section, atrazine was eluted with MeOH from the different fibers tested. The obtained recoveries were lower than 0.1% in both MIP and NIP fibers, which would not allow their use in the SPME of target analytes, and thus MAA:BetCl DES was discarded as a possible alternative to traditional solvents in the preparation of imprinted fibers. On the contrary, the use of hydrophobic DES yielded homogeneous solid monoliths. Therefore, the ability of the new DES-based fibers to rebind triazines in organic media was extensively investigated, and the results are presented in the following sections.

Selected DES and their individual components (L-menthol, thymol, camphor, MAA, and FA) were characterized by FT-IR spectroscopy, and their corresponding spectra are shown in Fig. S4–S7.

Figure S4 shows the infrared spectra of the optimal DES and its components (L-menthol and MAA). It can be observed that the intense peak at 1690 cm^−1^ in the MAA spectrum, corresponding to C=O stretching, is shifted to 1694 cm^−1^ in the infrared spectrum of DES, suggesting that the carboxylic acid moiety is involved in the establishment of hydrogen bonds during DES formation. In addition, the stretching of the O–H bond, observed in the infrared spectrum of MAA from ⁓3400 to ⁓2500 cm^−1^, is clearly affected, and the band becomes almost imperceptible in the infrared spectrum of DES, again indicating the interaction by hydrogen bonding between MAA and L-menthol. Similarly, the band at 3240 cm^−1^, corresponding to O–H vibration in the L-menthol spectrum, almost disappears in the spectrum of the obtained DES, thus confirming the formation of new hydrogen bonds. Similar bands and shifts are observed in the IR spectra of thymol:MAA- and L-menthol:FA-based DES compared to their individual components (Fig. S5 and S7, respectively), confirming the existence of new hydrogen bonds in both DES. Finally, regarding camphor:MAA-based DES (Fig. S6), the intense peak observed at 1738 cm^−1^ corresponding to the C=O stretching in camphor is shifted to 1732 cm^−1^, whereas the peak at 1690 cm^−1^ present in MAA spectra is shifted to 1693 cm^−1^ in the DES spectrum, indicating the formation of new hydrogen bonds.

### Rebinding experiments in hydrophobic DES-based imprinted fibers

Hydrophobic DES composed of L-menthol, thymol, or camphor as HBA and MAA as HBD were used as porogen/monomer for the preparation of both MIP and NIP fibers by thermal polymerization at different times (30, 60, 120 and 150 min). In parallel, fibers using toluene as porogen were also prepared for comparison purposes. The obtained fibers were immersed in a 1 mg L^−1^ ATZ standard solution in toluene (1.7 mL), and extraction was performed for 60 min under stirring. After a 15 min washing step with toluene (1.7 mL), the fibers were immersed in 225 µL methanol for 30 min under stirring for elution of ATZ. Elution fractions were analyzed by HPLC–DAD as described above (“Materials and methods” section).

**Fig. 1:**
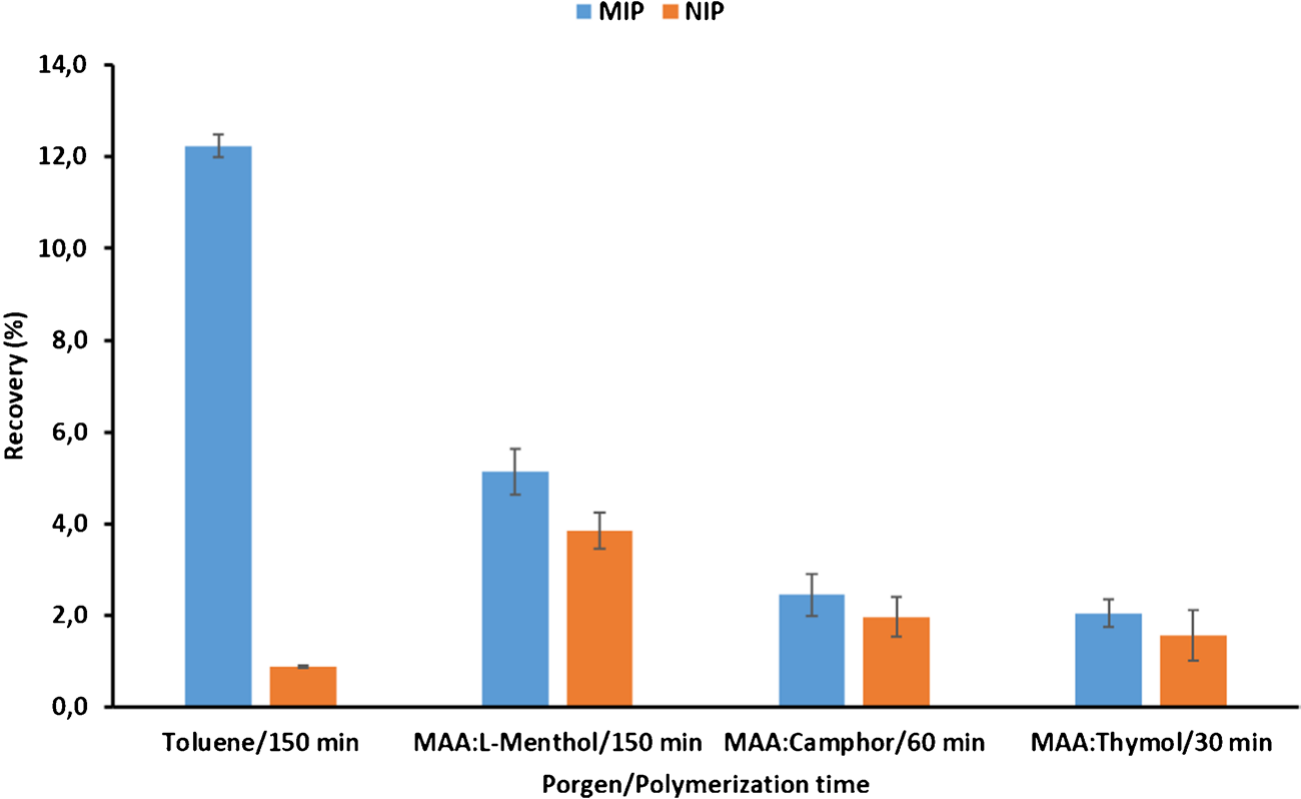
Recoveries obtained after SPME of atrazine standard solutions (1 mg L^−1^) in toluene using different porogens and polymerization time

Figure 1 shows the best recoveries obtained for the different fibers tested compared to the recoveries provided by the fibers prepared in toluene. Recoveries were calculated by comparing the obtained peak area measurements after SPME with those obtained by direct injection of a 1 mg L^−1^ ATZ standard solution in methanol:water (1:1, v/v). The results reveal that the performance of DES-based fibers is inferior to that provided by toluene-based fibers. However, the recoveries obtained are sufficient for the determination of target analytes in environmental samples, with the advantage of replacing a harmful solvent such as toluene with DES during MIP synthesis. Moreover, the retention in DES-based NIP fibers is also higher, suggesting that nonspecific interactions occur regardless of the DES used as porogen during polymerization. From this study, fibers prepared with MAA: L-menthol as porogen were selected for further study, as MIP fibers provided the highest recovery and, although nonspecific interactions occurred, the highest MIP/NIP recovery ratio suggested the presence of a larger number of imprinted binding sites.

**Fig. 2:**
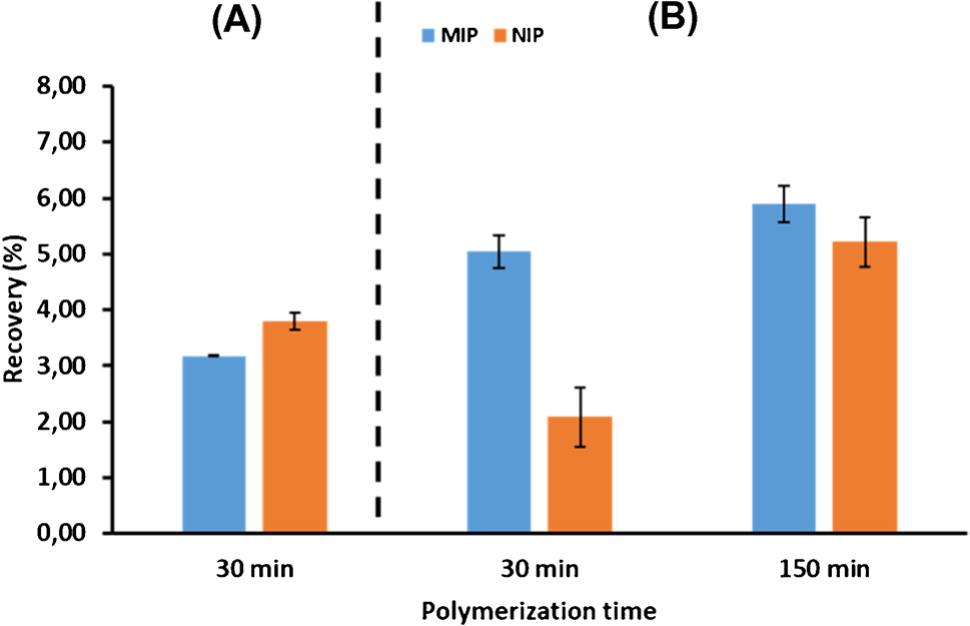
Recoveries obtained after SPME of atrazine standard solutions (1 mg L^−1^) in toluene using different fibers prepared (**A**) without MAA in the polymerization mixture and (**B**) using FA:L-menthol DES as porogen at two polymerization times (30 and 150 min)

The reason behind such large nonspecific interactions is unclear, but it might be attributed to the excess MAA used, since it was present both in the polymerization mixture and in the DES used. Therefore, a new set of fibers was prepared by eliminating MAA from the polymerization mixture (keeping MAA:L-menthol DES as porogen) or by replacing MAA with FA to prepare a new FA:L-menthol (1:1) DES to be used as porogen. These new fibers were used in the SPME of 1 mg L^−1^ ATZ standard solution in toluene according to the same protocol as described above. From this study, it was observed that fibers prepared with a polymerization mixture without MAA did not provide selective recognition (Fig. 2A). This result is consistent with the basis of molecular imprinting technology, where a large amount of functional monomer is required to stabilize the template:monomer complex during polymerization following the non-covalent approach. On the contrary, as shown in Fig. 2B, a significant reduction in the recoveries obtained by SPME was observed using NIP fibers (polymerization time: 30 min) prepared with FA:L-menthol DES, whereas the recoveries obtained using the corresponding MIP fibers were not negatively affected. This result confirms that an excess of MAA was, at least to some extent, responsible for the large nonspecific interactions observed using DES prepared with MAA as HBD. In addition, because polymerization time controls the degree of cross-linking of the polymer network and thus affects fiber porosity, a new set of fibers was prepared using a polymerization time of 150 min, and the results are also shown in Fig. 2B. As can be observed, although recoveries obtained by MIP fibers were improved, nonspecific interactions were also higher, and thus the resulting fibers were not suitable for the selective extraction of target analytes. Accordingly, fibers prepared using FA:L-menthol DES as porogen with a polymerization time of 30 min were selected for further studies.

### Cross-reactivity (selectivity) study

**Fig. 3:**
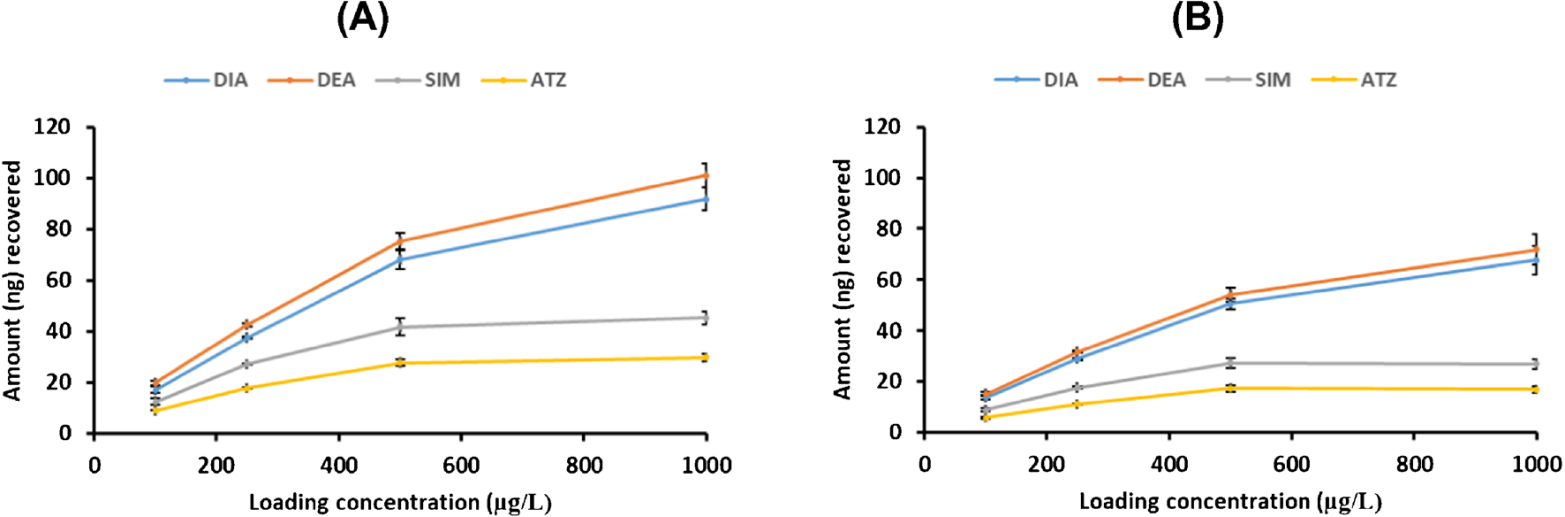
Amount (ng) of each triazine extracted at different loading concentrations by SPME using optimal MIP (**A**) and NIP (**B**) fibers

The ability of the new fibers to recognize other structurally related compounds was also studied by performing SPME of toluene solutions containing mixtures of four triazines (DIA, DEA, SIM, and ATZ). The concentration range studied was set from 100 to 1000 μg L^−1^ in order to estimate the capacity of the fibers as well as to evaluate possible competition between the different triazines for the binding sites present in the imprinted fibers. For purposes of comparison, parallel rebinding experiments were also performed for all selected triazines using the NIP fiber. Figure 3 shows the results obtained, expressed as nanograms recovered versus loaded concentration of each triazine in both MIP and NIP fibers. As can be observed, the amount (ng) of each triazine extracted by the MIP fiber (Fig. 3A) is higher than that extracted by the NIP fiber (Fig. 3B), demonstrating the existence of selective binding sites able to recognize the different triazines tested.

As can be observed, the retention of the smallest triazines (DIA and DEA) is favored in both MIP and NIP. Similar behavior has been observed using conventional MIPs and has been attributed to the ability of the smallest analytes to reach binding sites located in polymer micropores not easily accessible to the larger analytes [28, 30]. In addition, in order to demonstrate the selectivity of the new imprinted fibers, SPME of 1 mg L^−1^ isoproturon (IPN) solution in toluene was performed. IPN is an herbicide of the phenylurea family with a different chemical structure but similar size and polarity to that of ATZ (log P IPN = 2.32; log P ATZ = 2.2). The recoveries obtained for IPN using MIP and NIP were only 0.32% and 0.30%, respectively, demonstrating the selectivity provided by the new fibers.

In summary, it can be concluded that the proposed imprinted fibers were able to selectively extract triazines displaying sufficient capacity (⁓ 50 ng, depending upon the analyte) for environmental analysis and thus were further studied for the extraction of target analytes from real soil sample extracts.

### MI-SPME optimization (loading, washing, and elution conditions)

In order to reduce the observed nonspecific interactions, the loading, washing, and elution experimental conditions were optimized. Firstly, the loading time was optimized by immersing both MIP and NIP fibers in a triazine standard solution (1 mg L^−1^ of each analyte in toluene (1.7 mL), and extraction was performed for 15, 30, and 60 min under stirring. The results are shown in Fig. S8. As expected, the recoveries increased with loading time for both MIP and NIP. However, the ratio between the recoveries obtained using MIP fibers and those obtained using NIP fibers was improved at a loading time of 30 min, which was therefore selected as optimal and used in further experiments. The washing and elution times were similarly studied, but no significant differences were observed at the different times studied, so the washing and elution times were both set at 15 min.

**Fig. 4:**
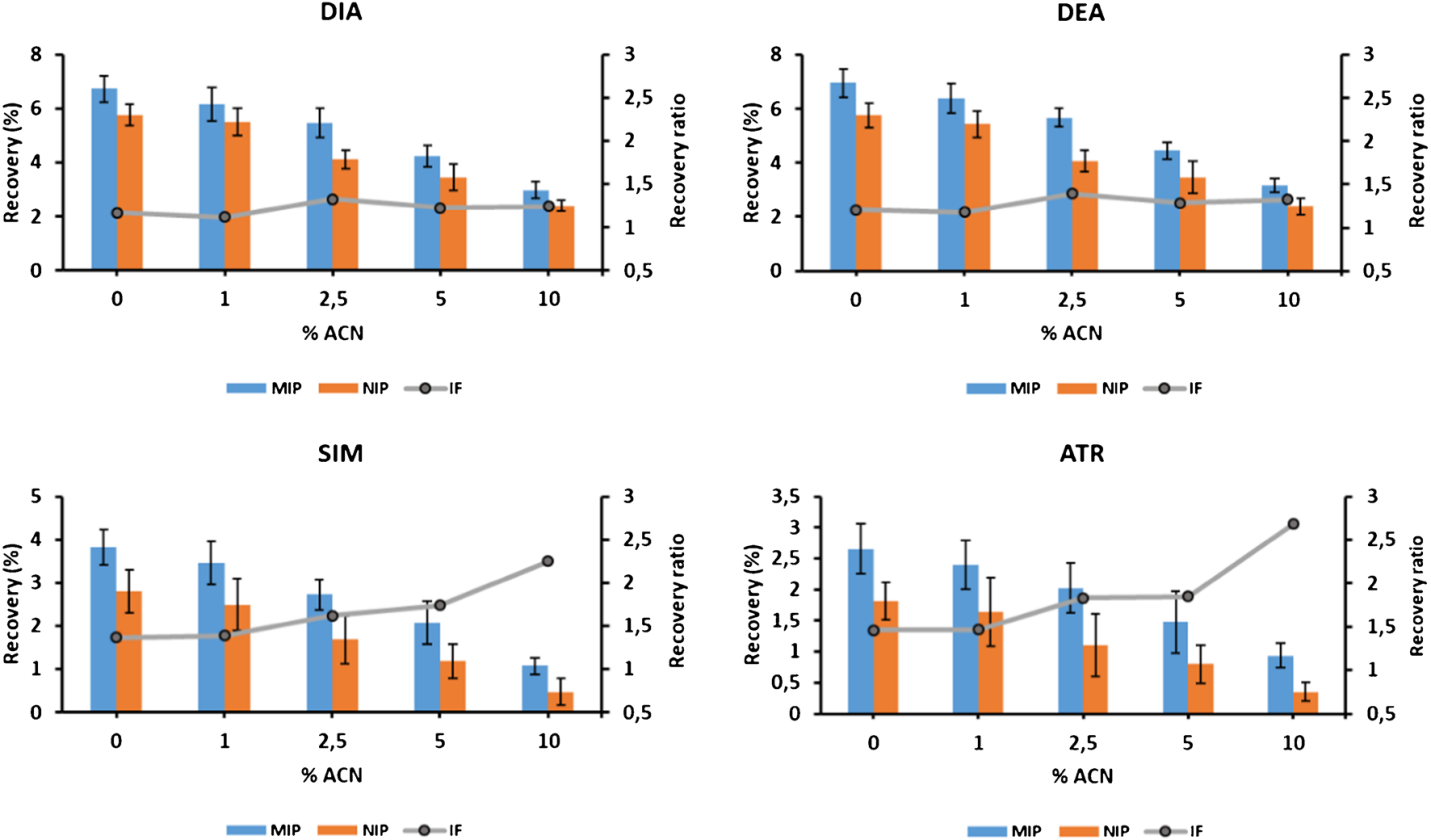
Effect of the amount of acetonitrile in the washing solution on the recoveries and MIP/NIP recovery ratios of target analytes after SPME of 1 mg L^−1^ solutions in toluene

Under the abovementioned optimal SPME conditions, different toluene:ACN mixtures were used as washing solvents in order to further reduce nonspecific interactions, and the results are shown in Fig. 4. It is clear that by increasing the amount of acetonitrile in the washing solution, a decrease in the recoveries obtained was observed for the four triazines under study (both in MIP and NIP fibers). However, at 2.5% ACN, a better MIP/NIP recovery ratio was obtained for all the analytes. A larger amount of ACN did not improve the recovery ratio for DIA and DEA, whereas a steady increase was observed for SIM and ATZ. However, despite this improvement for SIM and ATZ, the absolute recoveries were close to 1%, which is too low for environmental analysis. Accordingly, a washing step using a mixture of toluene:ACN (97.5:2.5, v/v) was considered optimal as it was able to remove nonspecific interactions to a greater extent while keeping recoveries in MIP fibers high enough for the extraction of target analytes from environmental samples.

### Analytical performance and application to the SPME of triazines from soil sample extracts

The proposed MI-SPME method was applied for the determination of triazines in soil sample extracts. Soil samples were spiked with a mixture of triazines at different concentration levels ranging from 0.01 to 0.25 µg g^−1^, and extracts were obtained as described above. MI-SPME was performed, and the analytes were determined by HPLC–DAD at 220 nm. Calibration graphs were constructed, and regression coefficients higher than 0.987 were obtained for all the analytes. The limits of detection (LODs), calculated as three times the standard deviation of the lower concentration, were in the range of 6.2–15.7 ng g^−1^, which is sufficiently low for the determination of triazines at environmentally relevant concentrations [31]. Table 2 shows the relative recoveries, and the corresponding standard deviations (SDs, *n* = 3), obtained from soil samples spiked with the analytes at three different concentration levels, along with the linear range, limits of quantification, and limits of detection. It can be observed that recoveries obtained from soil samples were comparable to those obtained for standards prepared in pure toluene, resulting in relative recoveries ranging from 75.7 to 120.1% but with slightly high SDs, especially for SIM and ATZ at the lower concentration level tested. However, it is important to point out that each extraction was performed with a different fiber from a different synthesis batch, and thus such SDs are also affected by the variability in the imprinted fiber synthesis. Nevertheless, it would be advisable to use the standard addition method for the determination of triazines by the proposed method.

**Table 2:** Relative recoveries (RR, %), and the corresponding SDs (*n* = 3), at three different concentration levels, linear range (ng g^−1^), limits of quantification (LOQ, ng g^−1^) and limits of detection (LOD, ng g^−1^) obtained from soil samples spiked with the analytes at different concentration levels after the use of the proposed MI-SPME method

		RR ± SD		Linear range	LOQ	LOD
	0.01 µg g^−1^	0.05 µg g^−1^	0.10 µg g^−1^			
DIA	113.0 ± 5.2	75.7 ± 12.9	90.4 ± 8.1	9.3–150	9.3	6.2
DEA	113.1 ± 7.2	78.2 ± 12.9	87.7 ± 7.4	14.0–150	14.0	8.1
SIM	115.0 ± 19.2	97.2 ± 10.0	90.1 ± 5.4	12.3–150	12.3	7.6
ATZ	120.1 ± 19.1	85.3 ± 7.9	87.7 ± 7.0	45.5–150	45.5	15.7

**Fig. 5:**
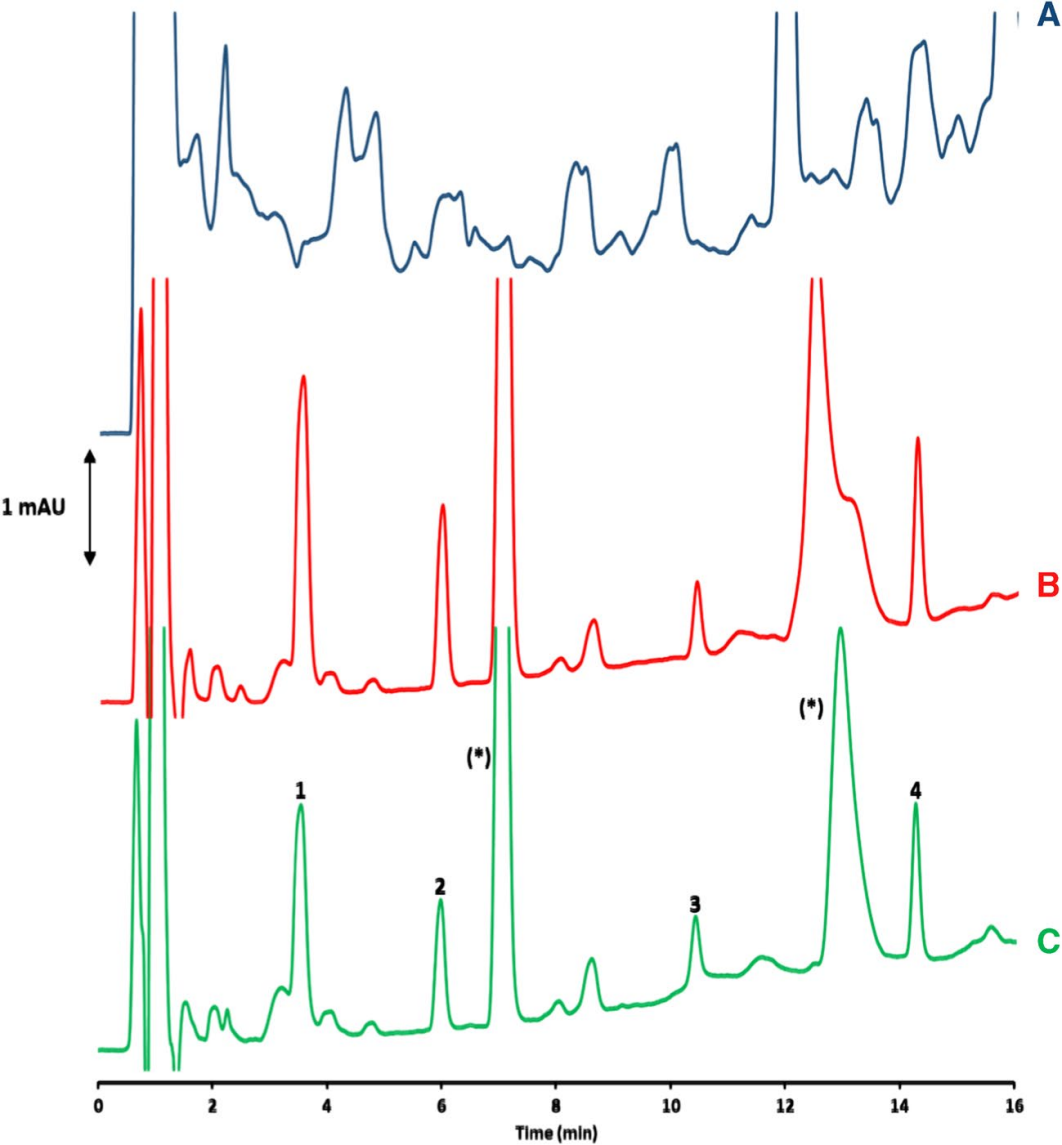
LC-UV chromatograms obtained at 220 nm for (**A**) a soil sample extract directly injected without any previous cleanup, (**B**) a 0.1 mg L^−1^ triazine standard solution after MI-SPME, and (**C**) a soil sample extract enriched with triazines at 0.05 µg g^−1^ concentration after MI-SPME. Peak numbers: (1) DIA; (2) DEA; (3) SIM; (4) ATZ; (*) unknown peaks. Chromatographic conditions: see Experimental section

Figure 5 shows the chromatograms obtained for a soil sample extract directly injected without previous cleanup (Fig. 5A), a triazine standard solution (0.1 mg L^−1^ each triazine) after MI-SPME (Fig. 5B), and a spiked (0.05 µg g^−1^ each triazine) soil sample extract after MI-SPME (Fig. 5C). As can be observed, the proposed MI-SPME procedure provides a high degree of selectivity for the extraction process, eliminating the co-extraction of sample matrix components. Such a high degree of clean-up enables the detection of target analytes at very low concentrations by ultraviolet (UV) detection. Moreover, the shape of the chromatograms obtained after MI-SPME for both standard solution and sample extract (at an equivalent concentration level) is quite similar, demonstrating the selectivity provided by the imprinted fibers. However, it is important to note that although a loss in fiber performance was not observed after 15 extractions (including real samples), two broad unknown peaks (labeled as (*) in Fig. 5) appeared in the chromatograms after MI-SPME of both standards and soil sample extracts. This result suggests the progressive degradation of the fibers, which could affect the accurate determination of target analytes if fibers were used more than 15 times.

Table S1 shows the relative recoveries, limits of detection, and limits of quantification (calculated as three and ten times the standard deviation of peak area measurements at the lower concentration level tested, respectively) of the present method in comparison with those of several other extraction methods for triazines in soil samples [32–37]. According to these figures, the proposed method is comparable to others, and thus it can be considered as an alternative to classical methods. Moreover, considering that the proposed method is based on a microextraction technique (SPME), it was worthwhile to evaluate its greenness using the Analytical Eco-Scale (ASE) tool [38]. The ASE is based on the assignment of penalty points to the different parameters and steps of an analytical process. The total penalty points are subtracted from 100, resulting in a score that qualitatively ranks the green analysis as excellent (> 75), acceptable (> 50), or inadequate (< 50). Table S2 shows the details of the penalty points assigned to the proposed method, which reaches a final score of 71, and it can thus be considered an acceptable green method. As can be observed, the highest penalty points are related to the organic solvents used, and therefore their replacement by greener solvents is desirable.

## Conclusions

In this work, a successful procedure for the preparation of imprinted fibers using a deep eutectic solvent as porogen for SPME has been proposed. The synthesis of imprinted fibers is easy to perform in the laboratory using basic equipment. The use of a DES as porogen made it possible to replace the conventional harmful solvents (i.e., toluene) typically used in molecular imprinting. The new greener fibers displayed similar properties to those synthesized using toluene as porogen, although with a more limited performance. Nevertheless, the capacity and selectivity provided were sufficient for its application to the SPME of triazines from soil sample extracts, reaching limits of detection comparable to those provided by classical methods.

This work demonstrates that pure DES can be used in molecular imprinting, thus opening new areas of research for obtaining imprinted polymers by greener synthesis procedures. However, some aspects, especially the unusually high nonspecific interactions, need to be further evaluated using other DES and templates. Furthermore, the synthesis of MIPs in different formats (i.e., beads) using DES as porogen should also be evaluated in order to demonstrate the general application of DES as greener solvents in molecular imprinting.

### Supplementary Information

Below is the link to the electronic supplementary material.

Supplementary file1 (DOCX 1.09 MB)

## Data Availability

The datasets generated during the current study are available from the corresponding author on reasonable request.
