# Supplementary material for: Evaluation of deep eutectic solvents in the synthesis of molecularly imprinted fibers for the solid-phase microextraction of triazines in soil samples
Source: Anal Bioanal Chem. 2024 Feb 3;416(6):1337–47. doi: 10.1007/s00216-024-05164-5 (PMC10861628; doi:10.1007/s00216-024-05164-5)
Supplement: Supplementary file 1 — Supplementary file1 (DOCX 1.09 MB) [file 216_2024_5164_MOESM1_ESM.docx]

**Supplementary Material**

**EVALUATION OF DEEP EUTECTIC SOLVENTS IN THE SYNTHESIS OF MOLECULARLY IMPRINTED FIBERS FOR THE SOLID-PHASE MICROEXTRACTION OF TRIAZINES IN SOIL SAMPLES**

Alexia Monnier, Myriam Díaz-Álvarez, Esther Turiel, Antonio Martín-Esteban*

Departamento de Medio Ambiente y Agronomía, INIA-CSIC, Carretera de A Coruña km. 7.5 28040 Madrid, Spain.

*Corresponding author: Tel: +34 913476821; fax: +34 913474008

E-mail address: amartin@inia.csic.es

Number of pages: 11; Number of figures: 8; Number of tables: 2

**Figure S1.-** Chemical structures of triazines and isoproturon

**
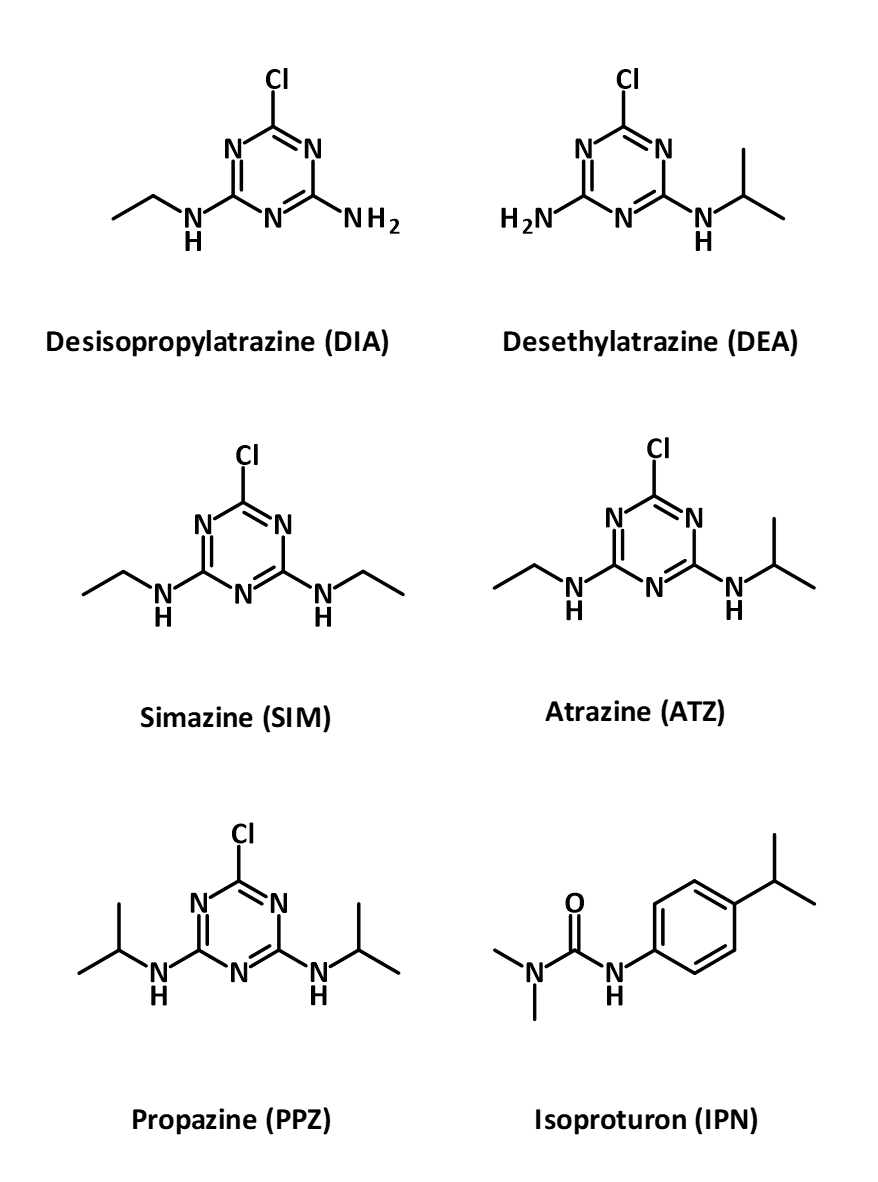
**

**Figure S2.-** Schematic diagram of the fiber preparation protocol. (1) Mark the position of the windows. (2) Burn the protective material and remove the ashes with water. (3) Fill the capillary with the polymerization mixture. (4) Close both ends and put in the oven at 65ºC for polymerization. (5) Cut the capillary on one edge of the window. (6) Dissolve the silica walls of the obtained fibers with a diluted NH_4_HF_2_ solution and then extract the template molecule with a methanol:acetic acid solution. (7) Fiber ready for use in SPME.

**
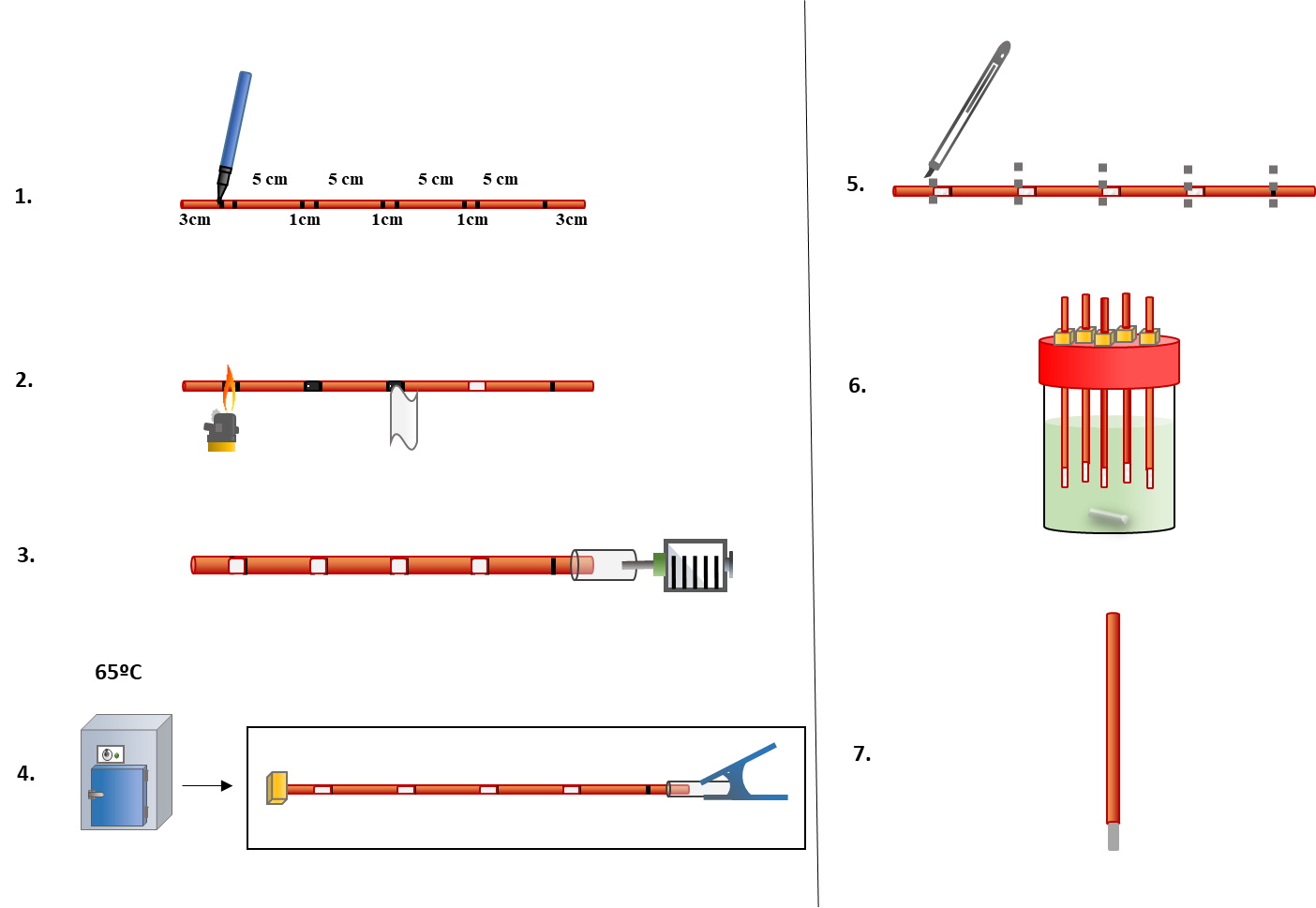
**

**Figure S3.-** Optical micrographs of fibers obtained using (A) MAA: BetCl and (B) MAA: L-menthol DES as porogen.

**
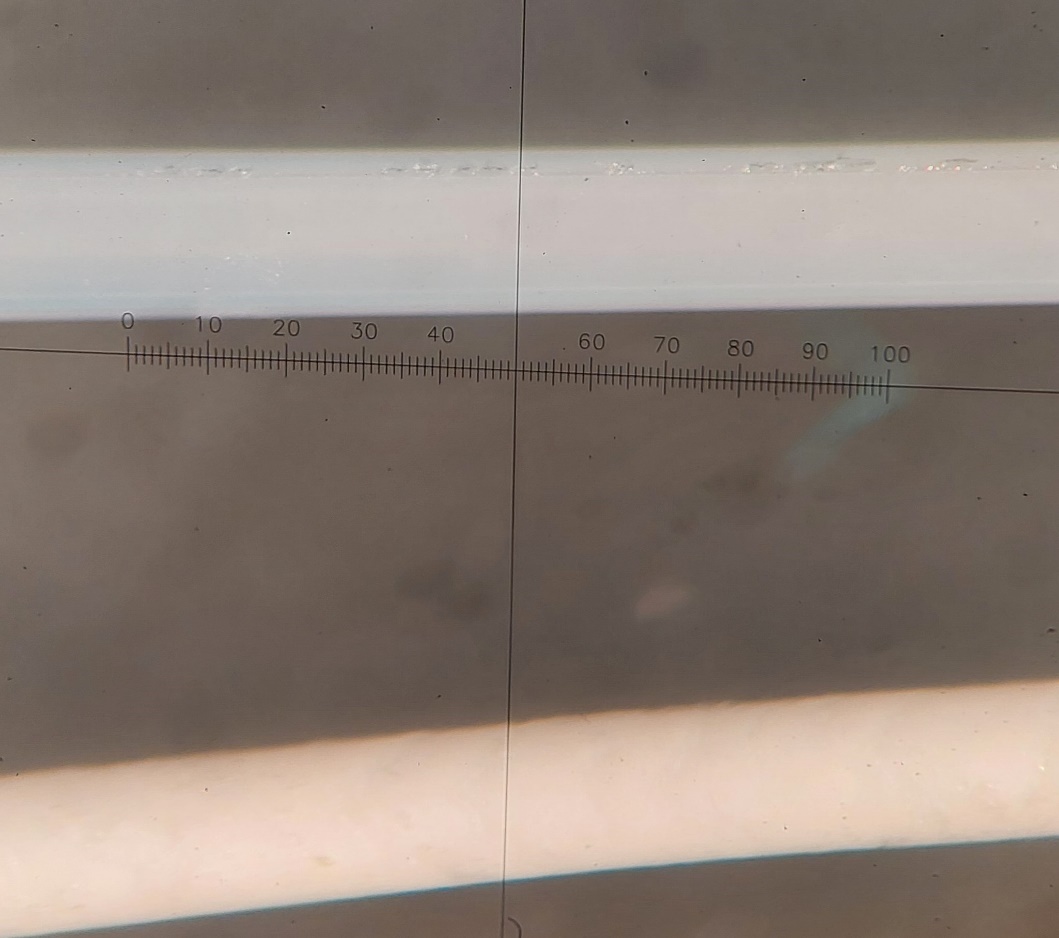
**

**(A)**

**(B)**

**Figure S4.-** FT-IR spectra of optimum DES and their components (L-menthol and MAA).


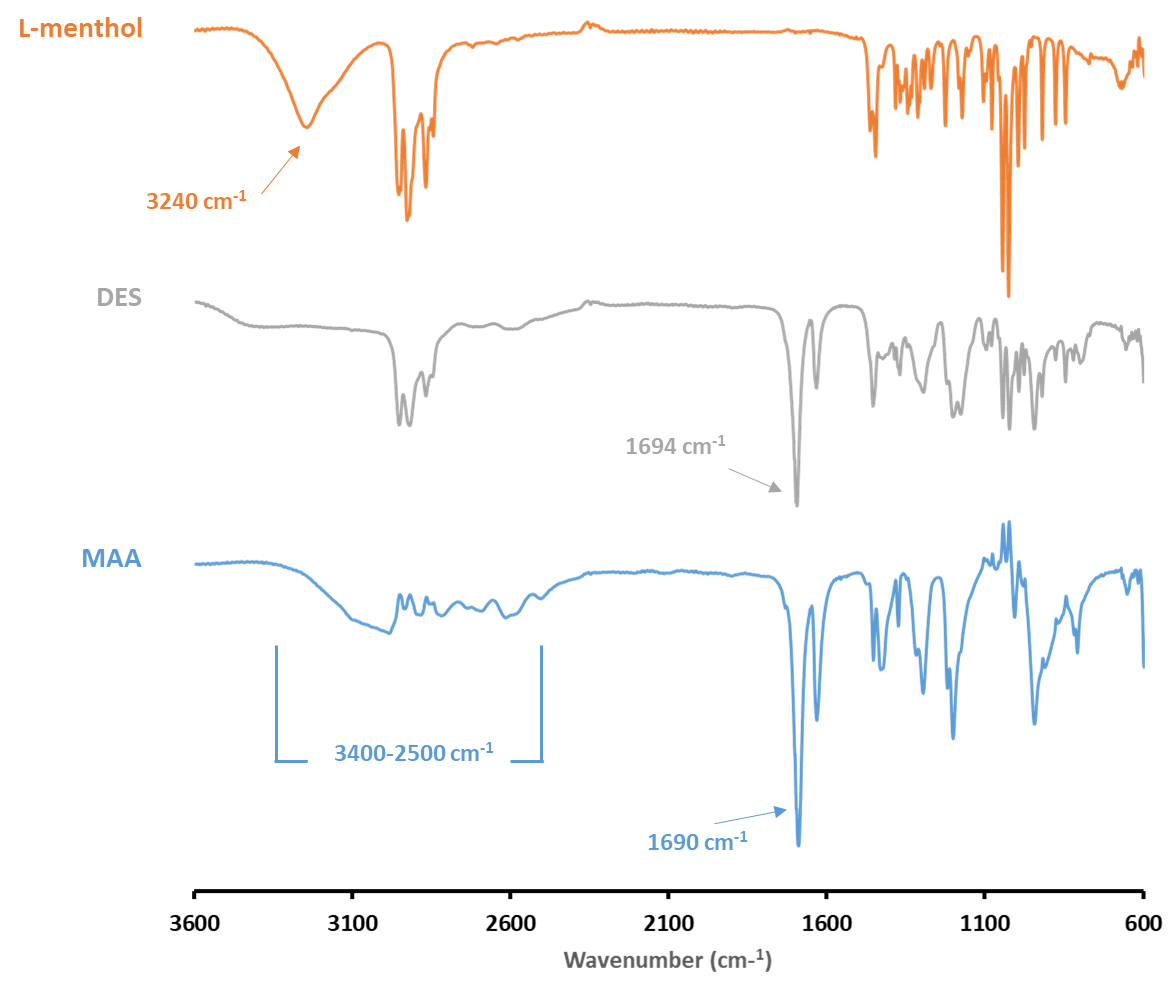


**Figure S5.-** FT-IR spectra of selected DES and their components (thymol and MAA).


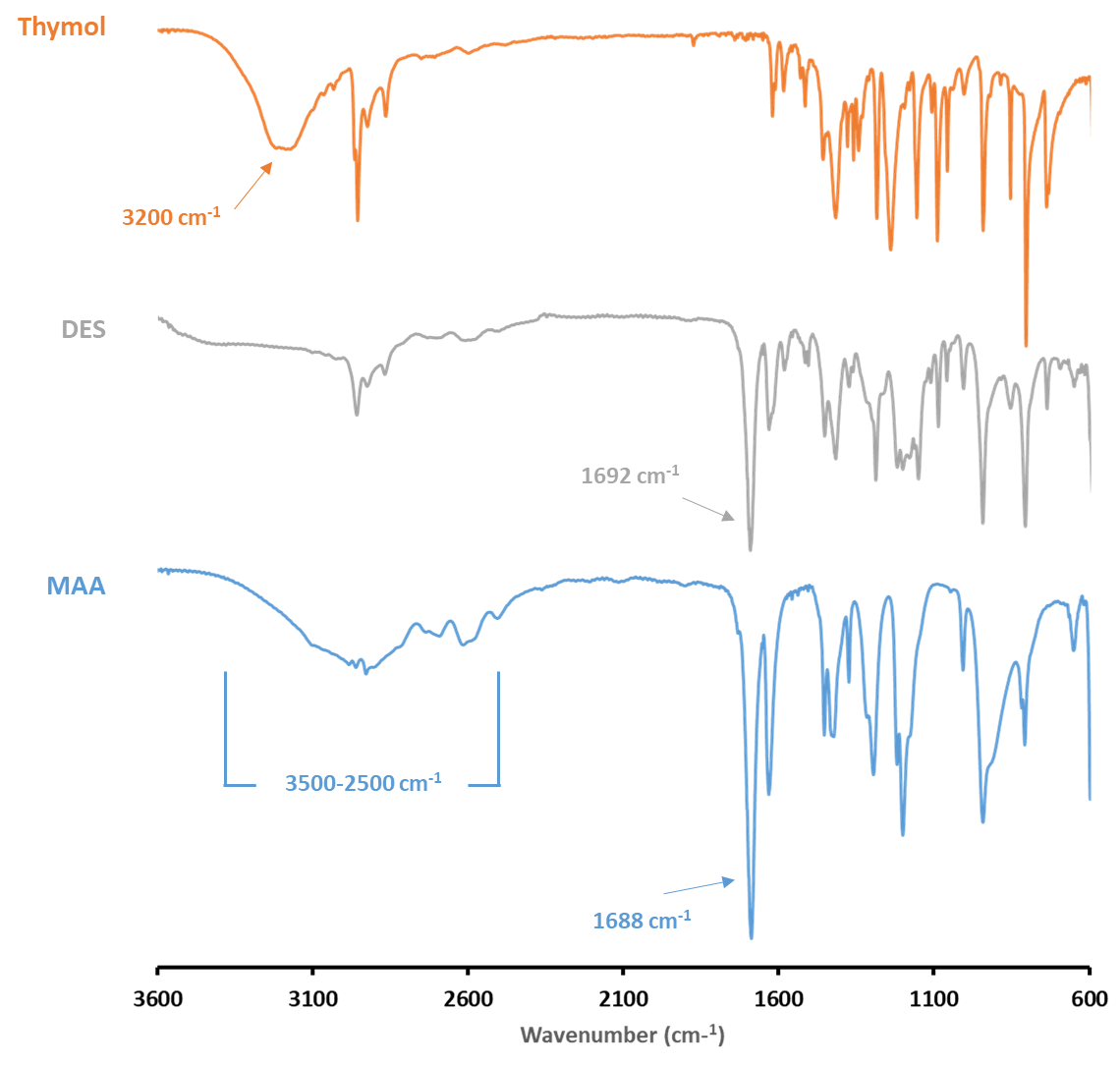


**Figure S6.-** FT-IR spectra of selected DES and their components (camphor and MAA).


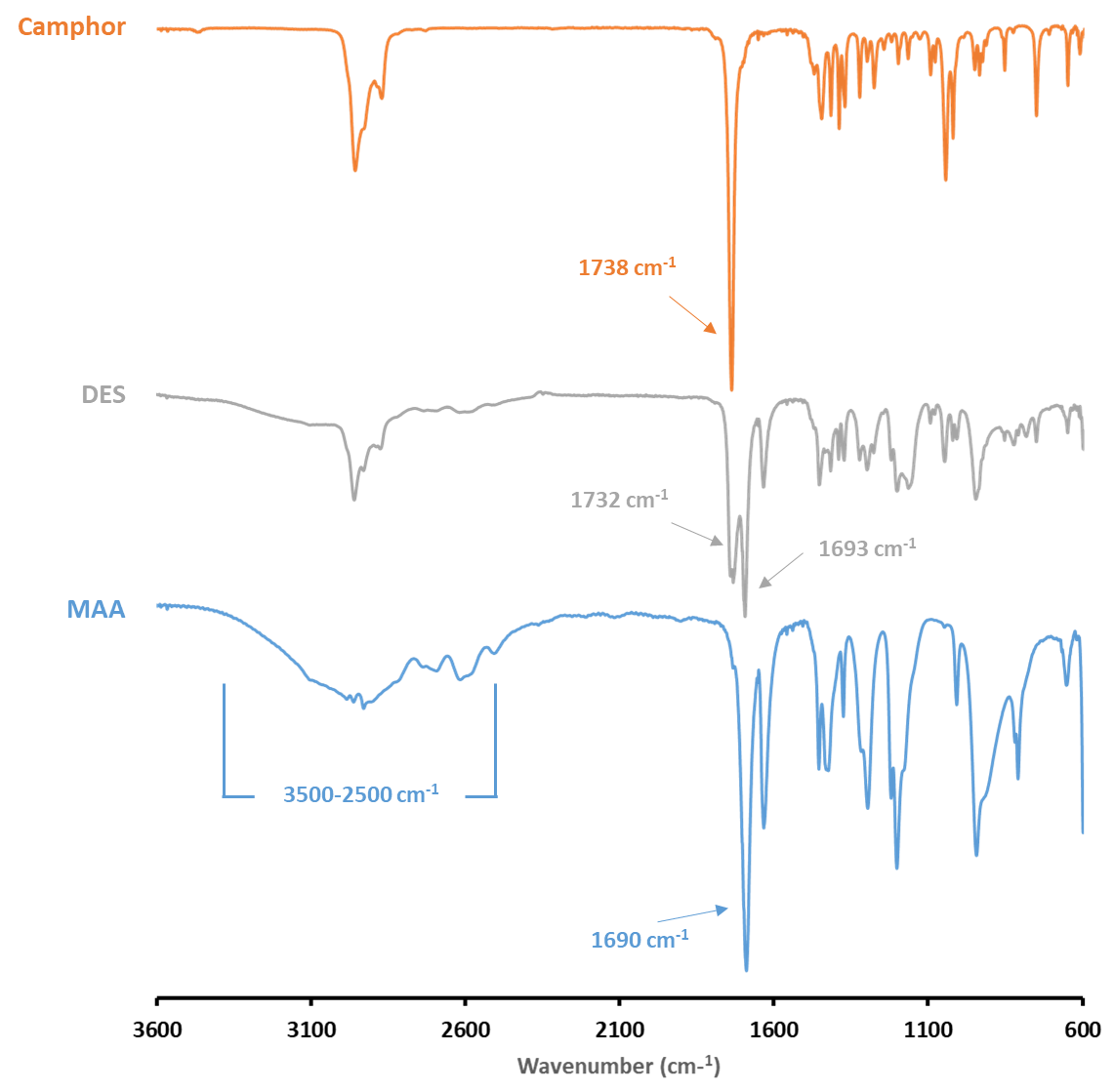


**Figure S7.-** FT-IR spectra of selected DES and their components (L-menthol and formic acid).


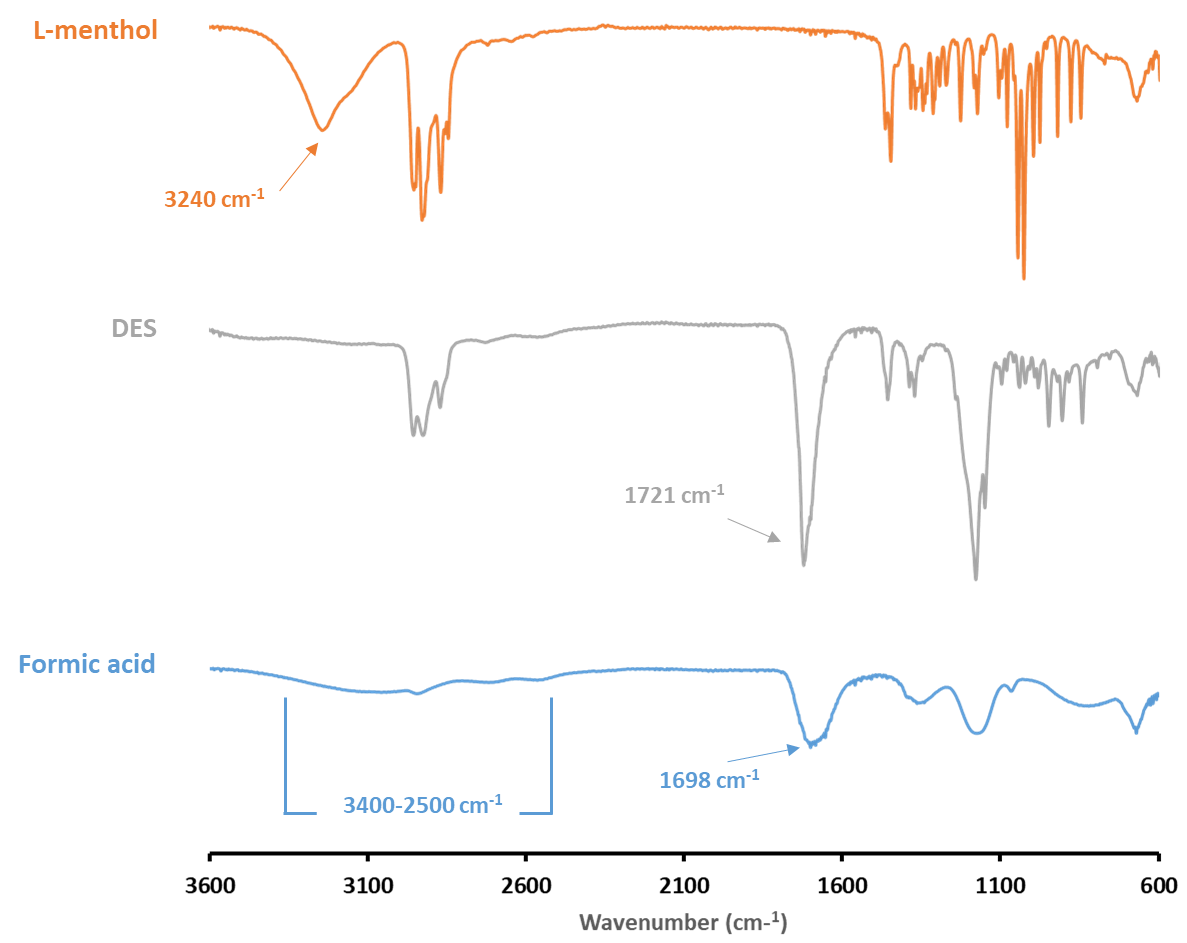


**Figure S8.-** Effect of loading time on the recoveries obtained in the SPME of a 1 mg L^-1^ mixture of triazines solution in toluene.


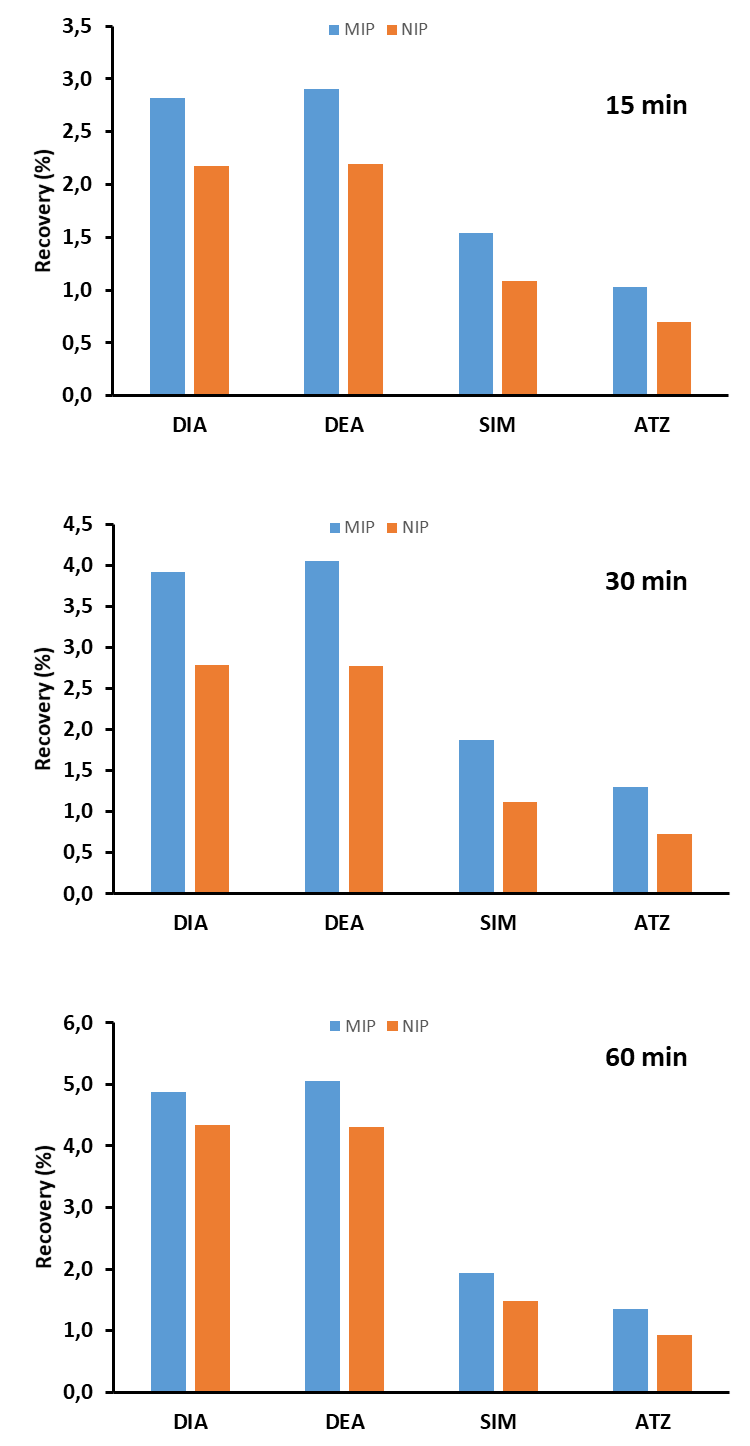


**Table S1.-** Comparison of the proposed method with other published methods for triazines determination in soil samples.

| **Sample preparation^*^** | **Analytical technique** | **Extraction solvent** | **Recovery (%)** | **LOD** | **LOQ** | **Ref** |
| --- | --- | --- | --- | --- | --- | --- |
| PLE | GC-MS/MS | Acetone (90 mL) | 80-120 | n.r. | 0.5-5 µg kg^-1^ | [32] |
| UAE | GC-TSD;GC-ECD | Acetone:n-hexane (20 mL, 2:1, v/v) | 70-90 | 5-30 ng g^-1^ | n.r. | [33] |
| MAE | HPLC-UV | Acetonitrile/ethylacetate/methanol (50 mL, 3:2:5,v/v/v) | 83–96 | 0.16-0.30 µg mL^-1^ | 0.50-1.0µg mL^-1^ | [34] |
| DMAME-CPP | HPLC-UV | Triton X-114 solution (3%, 10 mL) | 80-98 | 0.26-1.71 µg kg^-1^ | 0.86-5.69 µg kg^-1^ | [35] |
| MMI‐SPE | HPLC-DAD | Acetonitrile (10 mL) and Toluene (3 mL) | 13.6-50.2 | 0.1-3.0 ng g^-1^ | n.r. | [36] |
| SWE-MISPE | LC-MS/MS | Subcritical water | 78.9-101 | 0.4-3.3 µg kg^-1^ | n.r. | [37] |
| UAE-SPME | HPLC-DAD | Acetonitrile (20 mL) and Toluene (3 mL) | 75.7-97.2** | 6.2-15.7 ng g^-1^ | 9.3-45.5 ng g^-1^ | This work |

* PLE: pressurised liquid extraction; UAE: ultrasonic-assisted extraction; MAE: microwave-assisted extraction; DMAME-CPP: dynamic microwave-assisted micelle extraction and cloud point preconcentration; HFM-Protected-MI-MSPE: Hollow Fibre Membrane-Protected Molecularly Imprinted Microsolid-Phase Extraction; SWE-MISPE: Subcritical water extraction-molecularly imprinted solid-phase extraction; n.r.: not reported

** Relative recovery

**Table S2**. The penalty points for the proposed method according to the Analytical Eco-Scale tool [38].

| **Reagents** | |
| --- | --- |
| ***Penalty points*** | |
| Toluene: 3 mL | 6 |
| Acetonitrile: 20 mL | 8 |
| Methanol: 0.25 mL | 6 |
|  | Σ 20 |
| **Instruments** | |
| ***Penalty points*** | |
| HPLC-DAD | 0 |
| Sonication | 1 |
| Occupational exposure | 3 |
| Waste | 5 |
|  | Σ 9 |
|  |  |
| Total penalty points: 29 |  |
| **Analytical Eco-Scale total score: 71** |  |
